# Levodopa‐Carbidopa Intestinal Gel Improves Dyskinesia in Parkinson's Disease: Post Hoc Analysis from the COSMOS Study

**DOI:** 10.1002/mdc3.70222

**Published:** 2025-08-15

**Authors:** Alfonso Fasano, Cleanthe Spanaki, Tanya Gurevich, Robert Jech, Per Svenningsson, József Szász, Lydia Vela‐Desojo, Mihaela Simu, Lars Bergmann, Abdallah Saad, Juan Carlos Parra, Norbert Kovács

**Affiliations:** ^1^ Edmond J Safra Program in Parkinson's Disease and Morton and Gloria Shulman Movement Disorders Clinic, Toronto Western Hospital and Division of Neurology, UHN, Division of Neurology University of Toronto Toronto ON Canada; ^2^ Krembil Research Institute Toronto ON Canada; ^3^ Neurology Department University General Hospital of Heraklion PA.G.N.I Heraklion Greece; ^4^ Movement Disorders Unit, Neurological Institute Tel Aviv Medical Center, Tel Aviv University Tel Aviv Israel; ^5^ Department of Neurology and Center of Clinical Neurosciences, First Faculty of Medicine Charles University in Prague, and General University Hospital in Prague Prague Czech Republic; ^6^ Section of Neurology, Department of Clinical Neuroscience Karolinska Institutet Stockholm Sweden; ^7^ Faculty of Medicine GE Palade University of Medicine, Pharmacy, Science and Technology of Tîrgu Mureș, and Emergency Clinical County Hospital Mureș Tîrgu Mureș Romania; ^8^ Department of Neurology Hospital Universitario Fundación Alcorcón Alcorcón Spain; ^9^ Department of Neurology Victor Babes University of Medicine and Pharmacy Timisoara Romania; ^10^ AbbVie Inc. North Chicago IL USA; ^11^ Department of Neurology Medical School, University of Pécs Pécs Hungary

**Keywords:** dyskinesia, levodopa‐carbidopa intestinal gel, monotherapy, Parkinson's disease, observational studies

## Abstract

**Background:**

Dyskinesia is a debilitating complication of dopaminergic therapy in advanced Parkinson's disease.

**Objectives:**

To evaluate the effect of levodopa‐carbidopa intestinal gel (LCIG) on dyskinesia burden.

**Methods:**

This is a post hoc analysis of the retrospective, observational **CO**medication **S**tudy assessing **M**ono‐ and c**O**mbination therapy with levodopa‐carbidopa inte**S**tinal gel (COSMOS; NCT03362879). Change in dyskinesia was assessed by LCIG treatment group (monotherapy, daytime monotherapy, polytherapy), baseline dyskinesia duration (<4 vs. ≥4 hours), and dyskinesia severity (troublesome vs. non‐troublesome). Correlations between changes in dyskinesia and patient‐reported outcomes (Parkinson's Disease Questionnaire‐8 [PDQ‐8], Parkinson's Disease Sleep Scale‐2 [PDSS‐2], non‐motor symptoms scale [NMSS]) were assessed using Spearman correlation coefficients. The Unified Parkinson's Disease Rating Scale IV measured dyskinesia duration (Item 32), severity (Item 33), and pain (Item 34). Data were collected cross‐sectionally at a single study visit.

**Results:**

Over 50% (202/369) of LCIG‐treated patients experienced improvement in dyskinesia severity. Improvements in dyskinesia duration and severity were noted in all treatment groups. The proportion of patients with troublesome dyskinesia and amount of “Off” time significantly decreased from baseline to study visit, regardless of baseline dyskinesia burden (*P* < 0.01); dyskinesia duration improved only in the ≥4‐hour subgroup (*P* < 0.01). In the ≥4‐hour subgroup, dyskinesia duration correlated positively with PDQ‐8; dyskinesia severity correlated positively with PDQ‐8 and PDSS‐2; and dyskinesia pain correlated positively with PDQ‐8, PDSS‐2, and NMSS.

**Conclusion:**

LCIG led to reductions in dyskinesia severity, regardless of baseline dyskinesia burden. Dyskinesia duration improved in patients with high dyskinesia burden but not in those with low dyskinesia burden.

The current gold standard for treatment of Parkinson's disease (PD) is levodopa.^1^ However, as PD progresses and patients experience worsening motor and non‐motor symptoms, the ability to control PD symptoms with oral levodopa is reduced due to a narrowing therapeutic window, erratic gastric emptying, denervation of the striatum, and the short half‐life of oral levodopa.^2^, ^3^ To counter these issues, additional dopaminergic and non‐dopaminergic treatments are introduced to achieve symptomatic control.^4^ During periods of therapeutic ineffectiveness, referred to as “Off” time, patients experience the hallmark motor symptoms of PD, including tremors, bradykinesia, rigidity, “Off” dystonia, balance problems, and freezing of gait.^5^ Patients and care partners also report changes in quality of life and the patient's ability to engage in daily activities due to these motor fluctuations.^6^, ^7^ Therapeutic options such as dose fragmentation and adjunct therapies, including dopamine agonists and catechol‐O‐methyltransferase inhibitors, lead to more complex treatment regimens to control PD symptoms, which further increase pill burden and may reduce a patient's adherence to treatment.^8^

Higher dosing of levodopa can lead to dyskinesias,^3^, ^7^ which can result in severe discomfort, fatigue, pain, disability, exhaustion, and loss of autonomy.^7^ Levodopa‐induced dyskinesia is also associated with poor quality of life, social embarrassment, isolation, and higher healthcare costs.^6^, ^7^ In a US population‐based study, approximately 30% of patients with PD developed dyskinesia after receiving oral levodopa treatment for a median duration of 4 years; among these patients, nearly 30% experienced moderate or severe dyskinesia.^9^ There exists a considerable number of patients who may benefit from treatments aimed at improving dyskinesia.

Levodopa‐carbidopa intestinal gel (LCIG), also known as carbidopa‐levodopa enteral suspension, is a gel suspension of levodopa‐carbidopa (20 mg/mL and 5 mg/mL, respectively) used as a continuous infusion over 16 to 24 hours in patients with advanced PD.^10^, ^11^ LCIG provides continuous levodopa delivery via a percutaneous endoscopic gastrostomy with a jejunal extension tube and has been shown in clinical trials and real‐world studies to improve motor complications, including dyskinesia, among patients with advanced PD.^12^, ^13^, ^14^, ^15^

COSMOS (**CO**medication **S**tudy assessing **M**ono‐ and c**O**mbination therapy with levodopa‐carbidopa inte**S**tinal gel) was a large real‐world, multinational, retrospective, post‐marketing observational study of patients with advanced PD treated with LCIG in a routine clinical setting.^16^ The COSMOS study was the first to evaluate the real‐world use of LCIG as monotherapy or polytherapy. This study demonstrated the long‐term effectiveness of LCIG as a monotherapy option, showing improvements in motor symptoms and dyskinesia in a significant number of patients while maintaining a positive risk–benefit profile.^17^ Study data from the GLORIA clinical registry showed that improvements in dyskinesia with LCIG were overall greater in those with high vs. low duration of dyskinesia at baseline, suggesting that a greater burden of disease at baseline can lead to larger clinical improvements after treatment.^18^ In this post hoc analysis of data from the COSMOS study, we further examined improvements in dyskinesia and other motor symptoms in patients treated with LCIG based on the burden of dyskinesia at baseline.

## Methods

### Study Design and Treatment

This post hoc analysis included patients who participated in the COSMOS study (NCT03362879). The full methodology of the COSMOS study has been described elsewhere.^16^ In brief, the study enrolled patients who were diagnosed with PD and treated with LCIG for ≥12 months. Data were collected cross‐sectionally during a single study visit. All data before the study visit, including clinical assessment measures, were collected by retrospectively reviewing patient medical records from the time of the decision to start LCIG treatment until the study visit. Patients were included in the study if they received continuous LCIG treatment for ≥80% of the days in the preceding year and were treated by the same physician since initiation of LCIG treatment. Patients were excluded if they had enrolled in previous or concurrent clinical trials while using LCIG therapy or were unable to complete study questionaries. There were three treatment groups based on the patient's treatment regimens 12 months post‐initiation: LCIG monotherapy, LCIG daytime monotherapy, and LCIG polytherapy. LCIG monotherapy was defined as use of LCIG alone without any other PD medications; LCIG daytime monotherapy was defined as LCIG with additional evening/night PD therapy after daytime LCIG infusion; and LCIG polytherapy was defined as LCIG with additional PD therapy at any time during the day.^16^

### Assessments

This analysis examined the percentage of patients from the total population who showed improvement, no change, or worsening of dyskinesia severity from baseline (defined as before starting LCIG) to the study visit. These data were based on the Unified Parkinson's Disease Rating Scale (UPDRS) Part IV Item 33.^19^ Dyskinesia duration (Part IV Item 32) and dyskinesia severity at baseline and study visit were evaluated by type of LCIG treatment received (monotherapy, daytime monotherapy, or polytherapy). Dyskinesia duration and severity were also evaluated in the following subgroups: male and female patients and patients with younger (<50 years) and older age (≥50 years) at PD onset. Dyskinesia severity was classified as troublesome dyskinesia (TSD) or non‐troublesome dyskinesia (non‐TSD). TSD was defined as a score of ≥1 on the UPDRS Part IV Item 33; response options ranged from mildly (1), moderately (2), severely (3), or completely disabling dyskinesia (4). Non‐TSD was defined as no/not disabling dyskinesia (score of 0). Changes in dyskinesia duration and dyskinesia severity were measured from baseline to the study visit in different groups of patients who experienced improvements or no changes (grouped together), or worsening of motor PD symptoms. These symptoms included tremor, “Off” dystonia, gait impairment, balance problems, and freezing of gait. Patients reported the presence of these symptoms at baseline and indicated whether they improved, did not change, or worsened at the study visit.

Dyskinesia was further assessed in patient subgroups who experienced <4 hours and ≥4 hours of dyskinesia during the waking day before starting LCIG. Changes in dyskinesia were also evaluated based on the severity of dyskinesia at baseline and during the study visit (TSD and non‐TSD). The 4‐hour cutoff was selected for this analysis because the mean duration of dyskinesia at baseline was approximately 4 hours for the total population.^16^ This cutoff has also been used in previous subgroup analyses of the GLORIA registry, which categorized the <4‐hour cutoff and ≥4‐hour cutoff as representing low and high burden of dyskinesia, respectively.^18^ “Off” time (assessed by UPDRS Part IV Item 39) and the levodopa equivalent daily dose (LEDD)^20^ were also evaluated at baseline and at the study visit in subgroups of patients with <4 hours and ≥4 hours of dyskinesia duration at baseline.

Correlations were assessed between change in dyskinesia duration, dyskinesia severity, and dyskinesia pain (assessed using UPDRS Part IV Item 34) and change in patient‐reported outcomes (PROs; 8‐item Parkinson's Disease Questionnaire [PDQ‐8], Non‐Motor Symptoms Scale [NMSS], Parkinson's Disease Sleep Scale‐2 [PDSS‐2]) in patients with <4 hours and ≥4 hours of dyskinesia at baseline.

Safety assessments included the number of adverse events (AEs) that had a reasonable possibility of being related to LCIG.

### Statistical Analysis

Data collected during the study visit and from patient records were analyzed using descriptive statistics. Due to the study's retrospective design, several assessments had missing data, and no imputation was performed. Chi‐square tests and t‐tests were conducted to identify differences in baseline characteristics between patients with <4 hours and ≥4 hours of dyskinesia at baseline. Dyskinesia severity, as assessed by UPDRS Part IV item 33, was transformed into numerical values using the following scale: 0 = not, 1 = mildly, 2 = moderately, 3 = severely, and 4 = completely disabling. Spearman correlation coefficients (*r*) were used to analyze correlations between changes in dyskinesia (duration and severity) and changes in PROs. Wilcoxon tests were conducted to assess the differences in outcomes before the initiation of LCIG compared with those recorded at the study visit. Statistical analyses were performed using SAS version 9.4 or higher (SAS Institute, Cary, NC).

## Results

### Patients

A total of 409 patients were included in the COSMOS study.^16^ Characteristics of the total population at baseline have been previously published and were generally comparable across LCIG treatment groups.^16^ Among the 268 patients with dyskinesia at baseline, 139 had a dyskinesia duration of <4 hours, and 129 had a dyskinesia duration of ≥4 hours (Table 1). The baseline characteristics of these two subgroups were generally similar. However, patients with dyskinesia for ≥4 hours compared with those with <4 hours had a longer duration of PD (mean 13.6 vs. 11.6 years; *P* < 0.01), and a longer time from PD diagnosis to the onset of motor fluctuations (7.8 vs. 6.5 years; *P* < 0.01) and to wearing off (7.9 vs. 6.5 years; *P* < 0.01). The mean (SD) duration of LCIG treatment was 30.9 (19.0) months in patients with <4 hours of dyskinesia and 36.1 (21.3) months in patients with ≥4 hours of dyskinesia. Following the initiation of LCIG, no patients needed to undergo deep brain stimulation (DBS). However, three patients had previously undergone DBS before starting LCIG and had remained on DBS therapy at the time of the study visit.

**TABLE 1:** Baseline demographics and disease characteristics

Characteristic	<4 hours	≥4 hours	*P* value
Male sex, *n* (%)	94 (67.6) n = 139	88 (68.2) n = 129	NS
Age, y	66.3 (8.0) n = 135	65.8 (8.0) n = 128	NS
Patients receiving LCIG monotherapy,^a^ *n* (%)	46 (35.9) n = 128	39 (31.0) n = 126	NS
Disease duration, y	11.6 (5.5) n = 136	13.6 (5.0) n = 128	<0.01
Morning akinesia, *n* (%)	98 (71.5) n = 137	97 (76.4) n = 127	NS
Wearing off, *n* (%)	127 (92.7) n = 137	123 (96.1) n = 128	NS
Time from PD diagnosis to motor fluctuation onset, y	6.5 (3.3) n = 134	7.8 (3.8) n = 125	<0.01
Time from PD diagnosis to dyskinesia, y	8.0 (3.6) n = 87	8.7 (4.3) n = 124	NS
Time from PD diagnosis to morning akinesia, y	6.9 (3.8) n = 94	7.7 (3.4) n = 95	NS
Time from PD diagnosis to wearing off, y	6.5 (3.2) n = 125	7.9 (3.8) n = 119	<0.01
LEDD, mg	1525.6 (1118.2) n = 118	1646.0 (1213.5) n = 118	NS

*Note*: Data reported as mean (SD) unless otherwise specified.

Abbreviations: LCIG, levodopa‐carbidopa intestinal gel; LEDD, levodopa equivalent daily dose; NS, not significant; PD, Parkinson's disease; SD, standard deviation.

^a^ 12 months after LCIG initiation.

### Efficacy

#### Improvements in Dyskinesia and Motor Symptoms with LCIG

**Figure 1:**
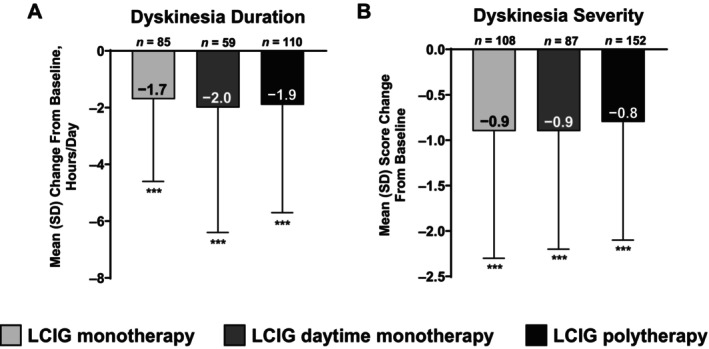
Change in (A) dyskinesia duration and (B) dyskinesia severity among patients who received LCIG monotherapy, LCIG daytime monotherapy, or LCIG polytherapy. LCIG, levodopa‐carbidopa intestinal gel; SD, standard deviation. ***P < 0.001 vs. baseline.

After starting LCIG treatment, 54.7% (n = 202) of patients experienced an improvement in dyskinesia severity whereas 33.1% (n = 122) and 12.2% (n = 45) experienced no change or worsening of dyskinesia severity, respectively. The duration and severity of dyskinesia improved from baseline to study visit, regardless of whether patients received LCIG as monotherapy, daytime monotherapy, or polytherapy (all *P* < 0.001; Fig. 1).

**Figure 2:**
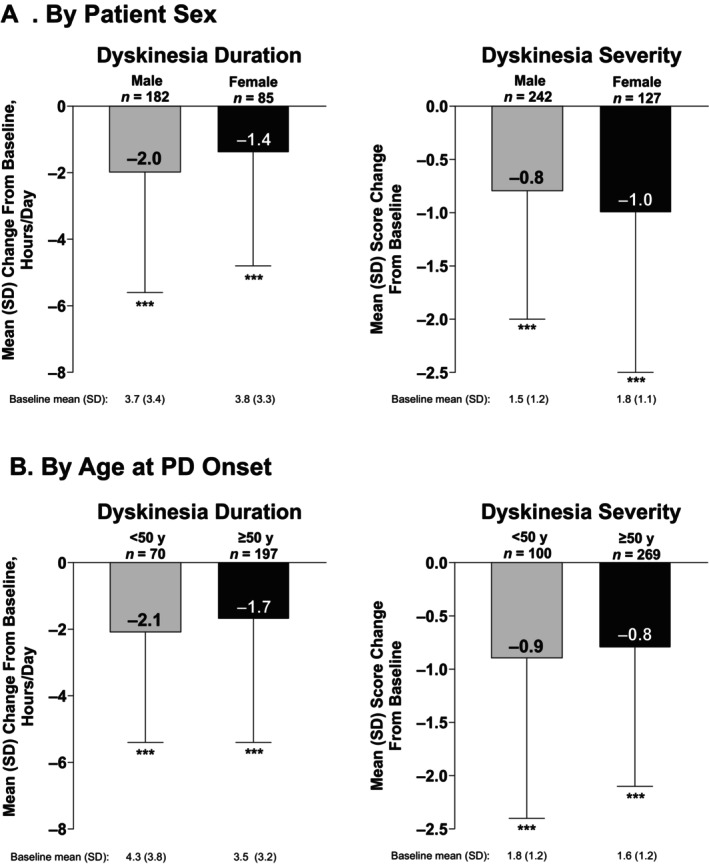
Change in dyskinesia duration and dyskinesia severity with LCIG treatment by (A) patient sex and (B) age at PD onset. PD, Parkinson's disease; LCIG, levodopa‐carbidopa intestinal gel; SD, standard deviation; y, years. ***P < 0.001 vs. baseline.

Dyskinesia duration and severity significantly improved from baseline to study visit in male and female patients and patients with younger (<50 years) and older age (≥50 years) at PD onset (all *P* < 0.001; Fig. 2).

Among patients treated with LCIG, the duration and severity of dyskinesia improved numerically, regardless of whether PD motor symptoms improved or worsened (Table 2). Patients receiving LCIG treatment who had gait impairments that improved or did not change experienced a significantly greater reduction in dyskinesia duration from baseline compared with those with worsened gait impairments (*P* < 0.05). Similarly, patients with “Off” dystonia and freezing of gait that improved or did not change with LCIG treatment experienced a significantly greater reduction from baseline in dyskinesia severity compared with patients who experienced worsening in “Off” dystonia or freezing of gait (*P* < 0.01 and *P* < 0.05, respectively).

**TABLE 2:** Change from baseline to study visit in dyskinesia duration and severity by change in PD motor symptom

Change in PD motor symptom^a^	Dyskinesia	
	Duration, h, mean (SD)^b^	Severity, mean (SD)^c^
Tremor		
Improved/not changed	−1.8 (3.5)^d^	−0.9 (1.3)^e^
Worsened	−2.1 (3.8)^f^	−0.5 (1.1)^g^
Dystonia		
Improved/not changed	−1.8 (3.5)^h^	−0.9 (1.3)** ^,i^
Worsened	−1.7 (3.4)^j^	−0.3 (1.3)^k^
Gait impairment		
Improved/not changed	−2.0 (3.6)* ^,l^	−0.9 (1.3)^m^
Worsened	−0.9 (3.4)^n^	−0.6 (1.3)^o^
Balance problems		
Improved/not changed	−1.7 (3.5)^p^	−0.9 (1.3)^q^
Worsened	−2.1 (3.9)^r^	−0.7 (1.4)^s^
Freezing of gait		
Improved/not changed	−1.9 (3.6)^t^	−0.9 (1.3)* ^,u^
Worsened	−1.3 (3.2)^v^	−0.4 (1.4)^w^

*Note*: ^a^As assessed by patients reporting the presence of individual symptoms at baseline and their change as improved, not changed, or worsened. ^b^As assessed by UPDRS Part IV Item 32. ^c^Data represent change in UPDRS Part IV Item 33 score. ^d^n = 244. ^e^n = 326. ^f^n = 20. ^g^n = 30. ^h^n = 325. ^i^n = 314. ^j^n = 26. ^k^n = 39. ^l^n = 214. ^m^n = 281. ^n^n = 51. ^o^n = 79. ^p^n = 200. ^q^n = 257. ^r^n = 65. ^s^n = 99. ^t^n = 232. ^u^n = 304. ^v^n = 31. ^w^n = 47.

Abbreviations: h, hours; PD, Parkinson's disease; SD, standard deviation; UPDRS, Unified Parkinson's Disease Rating Scale.

*P < 0.05; **P < 0.01 vs. patients with symptoms that worsened.

**Figure 3:**
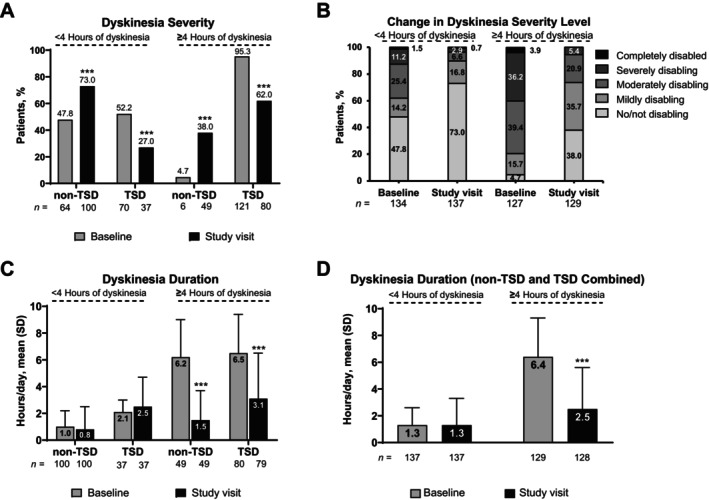
Change in dyskinesia severity and duration among patients with <4 or ≥4 hours of dyskinesia at baseline. SD, standard deviation; TSD, troublesome dyskinesia. ***P < 0.001 vs. baseline.

From baseline to study visit, LCIG led to a significant decrease in the proportion of patients with TSD among both subgroups of patients with <4 hours and ≥4 hours of dyskinesia at baseline (*P* < 0.001; Fig. 3A,B). In patients with ≥4 hours of dyskinesia at baseline, a significant decrease in dyskinesia duration was observed from baseline to study visit (*P* < 0.001; Fig. 3C,D). The duration of dyskinesia remained relatively stable from baseline to study visit in patients with <4 hours of dyskinesia at baseline, regardless of whether they had TSD or non‐TSD.

**Figure 4:**
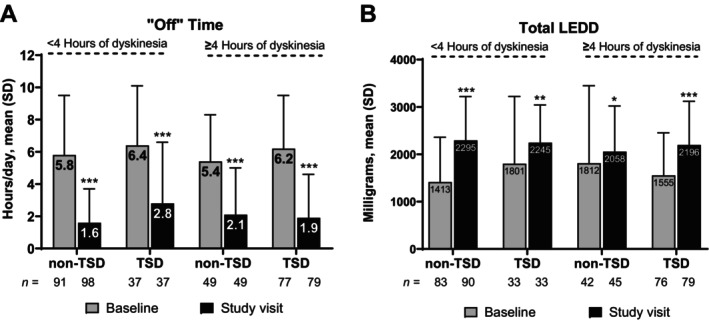
(A) “Off” time and (B) Total LEDD among patients with <4 and ≥4 hours of dyskinesia at baseline. LEDD, levodopa equivalent daily dose; SD, standard deviation; TSD, troublesome dyskinesia. *P < 0.05; **P < 0.01; ***P < 0.001 vs. baseline.

“Off” time decreased significantly from baseline to study visit among patients with <4 and ≥4 hours of dyskinesia at baseline, regardless of whether the dyskinesia was TSD or non‐TSD (all *P* < 0.001; Fig. 4A). Notably, a significant increase in mean total LEDD was observed from baseline to study visit in both subgroups, regardless of whether the type of dyskinesia was TSD or non‐TSD (all *P* < 0.05; Fig. 4B).

#### Correlations between Dyskinesia and PROs

In patients with ≥4 hours of dyskinesia, significant and weak positive correlations were observed between change in dyskinesia duration and PDQ‐8 scores (*r* = 0.2873; *P* < 0.01); significant and weak positive correlations were also observed between change in dyskinesia severity and PDQ‐8 scores (*r* = 0.2608; *P* < 0.01) and PDSS‐2 scores (*r* = 0.3624; *P* < 0.001; Table 3). There were significant, albeit weak, positive correlations between change in dyskinesia pain and PDQ‐8 scores (*r* = 0.3268; *P* < 0.001), PDSS‐2 scores (*r* = 0.3770; *P* < 0.001), and NMSS scores (*r* = 0.3040; *P* < 0.001) for the subgroup with ≥4 hours of dyskinesia. In patients with <4 hours of dyskinesia at baseline, a significant positive correlation was observed between change in dyskinesia duration and NMSS scores, but the strength of this correlation was very weak (*r* = 0.1704; *P* < 0.05). There were no other correlations between dyskinesia duration, severity, and pain and assessed PROs in the <4 hours of dyskinesia subgroup.

**TABLE 3:** Correlations of changes in dyskinesia with PROs in patients with <4 or ≥4 hours of dyskinesia at baseline and in the total patient population

Correlations between dyskinesia duration and PROs	Spearman correlation coefficient (*r*)		
	<4 hours	≥4 hours	Total
PDQ‐8 summary index	0.0751	0.2873**	0.1436*
PDSS‐2 total score	0.1423	0.1948	0.1626**
NMSS total score	0.1704*	0.2069	0.2156***
Correlations between dyskinesia severity and PROs			
PDQ‐8 summary index	0.1346	0.2608**	0.1852***
PDSS‐2 total score	0.1299	0.3624***	0.2057***
NMSS total score	0.1199	0.1941*	0.1766***
Correlations between dyskinesia pain and PROs			
PDQ‐8 summary index	0.1398	0.3268***	0.2185***
PDSS‐2 total score	0.1314	0.3770***	0.2252***
NMSS total score	0.0249	0.3040***	0.1351**

*Note*: Both patients with TSD and non‐TSD were included in each subgroup.

Abbreviations: NMSS, Non‐Motor Symptoms Scale; PDQ‐8, Parkinson's Disease Questionnaire‐8; PDSS‐2, Parkinson's Disease Sleep Scale‐2; PRO, patient‐reported outcome; TSD, troublesome dyskinesia.

* *P* < 0.05;

** *P* < 0.01;

*** *P* < 0.001.

### Safety

The most commonly reported AEs were stoma site infection, device malfunction, and dyskinesias.^16^ Dyskinesia was reported as an AE in nine patients, all of whom were receiving ≤16 hours of LCIG infusion per day.

## Discussion

Findings from this post hoc analysis of data from the COSMOS study showed that a large proportion (55%) of patients with dyskinetic fluctuations treated with LCIG experienced an overall improvement in dyskinesia severity. Importantly, we observed reductions in the duration and severity of dyskinesia from baseline, regardless of whether LCIG was administered as monotherapy, daytime monotherapy, or polytherapy. These findings support the use of LCIG as a therapeutic option to improve dyskinesia while reducing pill burden. These results also highlight the importance of continuous drug delivery in providing stable levodopa plasma levels during the waking day, which aids in attenuating peak levodopa levels^21^ and thereby reducing TSD and improving patient mobility. Our findings are also consistent with those from previous phase 3 randomized studies that showed the effect of continuous levodopa delivery with LCIG in reducing TSD.^14^, ^22^

We conducted analyses to determine if treatment with LCIG consistently improved dyskinesia across different subgroups, specifically in patients who may present with more severe dyskinesia, such as female patients and patients with young‐onset PD.^9^, ^23^ Our findings show that both male and female patients and patients with PD onset at age < 50 and ≥ 50 years experienced improvements in dyskinesia after initiation of LCIG.

Interestingly, patients treated with LCIG who experienced improvements or no change in gait also experienced significantly greater improvements in dyskinesia than patients with worsening of gait. This finding is particularly relevant as severe levodopa‐induced dyskinesia can have a substantial negative impact on gait.^24^ Similarly, patients who experienced improvements or no change in dystonia also showed significantly greater reductions in the severity of dyskinesia compared with those whose dystonia worsened. This finding may have been attributed to patients experiencing less “Off” time following LCIG treatment and, consequently, less frequent and/or severe dystonia.

The effects of LCIG on dyskinesia and “Off” time were examined in a subgroup analysis. Patients who had ≥4 hours of dyskinesia at baseline showed significant reductions in the daily duration of dyskinesia and “Off” time, regardless of whether they had TSD or non‐TSD. They also experienced significant improvements in the severity of dyskinesia. Patients in the <4‐hour subgroup showed improvements in dyskinesia severity and “Off” time. However, they did not experience improvements from baseline in the daily duration of dyskinesia, which may have been attributed to the fact that the mean dyskinesia duration at baseline was already quite low at 1.3 hours. Furthermore, these results suggest that improvements in dyskinesia may likely be greater in those with high vs. low burden of dyskinesia. These findings are consistent with a similar subgroup analysis performed by Poewe et al,^18^ which found that dyskinesia improved to a greater extent in those with high vs. low burden of dyskinesia at baseline.

Patients in this study had a significant increase in LEDD from baseline to study visit. Although increasing the dose of levodopa is a significant risk factor for levodopa‐induced dyskinesia,^25^ we observed overall improvements in dyskinesia from baseline. These results are likely explained by the use of LCIG to provide stable, continuous delivery of levodopa, which might avoid pulsatile stimulation of dopamine receptors and decrease the risk of peak‐dose dyskinesia, as discussed in previous reports.^3^, ^21^ Alternatively, the lack of levodopa peaks could have resulted in fewer or no dyskinesias in patients with diamond‐shaped dyskinesias at baseline compared with those patients with square‐wave types, as recently discussed by Gupta et al.^26^

The observed improvements in dyskinesia with LCIG had positive correlations with PROs, highlighting the adverse effects of dyskinesia on health‐related quality of life, non‐motor symptoms, and sleep. Among patients who experienced ≥4 hours of dyskinesia at baseline, the strongest correlations with PROs were observed with dyskinesia‐related pain, which underlines the profound impact that pain has on quality of life and emphasizes the need for treating dyskinetic pain. Notably, patients with higher vs. lower duration of dyskinesia at baseline had stronger correlations between changes in dyskinesia and all assessed PROs. These findings may suggest that symptoms must reach a certain threshold before improvements are translated into enhanced quality of life and perceived by patients to be meaningful. The correlation between dyskinesia and health‐related quality of life observed here is consistent with results from a separate study where a positive but weak correlation was found between PDQ‐8 summary index scores and dyskinesia, as measured by the Unified Dyskinesia Rating Scale (UDysRS).^27^ Comparatively, in another study that evaluated LCIG in patients with a higher burden of dyskinesia at baseline, moderate positive correlations were observed between dyskinesia and health‐related quality of life.^28^

The safety results were consistent with the known safety profile of LCIG. Dyskinesia was reported as an AE in nine (2.2%) patients in this study. It is important to note that dyskinesia is a common side effect of dopaminergic treatments including LCIG. Complex/atypical dyskinesias have been associated with LCIG treatment and may impair therapy, potentially leading to discontinuation.^29^ At the same time, real life clinical observations suggest that in parallel with predictable reduction in motor fluctuations, dyskinesias might evolve and change their profile during LCIG treatment (eg, a decrease in severe/complex dyskinesias could be associated with a slight increase in the duration of mild/moderate dyskinesias).^30^, ^31^ Therefore, it is essential to identify specific patient profiles that may respond favorably, unfavorably, or not at all to the anti‐dyskinetic effects of LCIG. This will ensure the safe selection of patients and raise clinicians’ awareness of the true anti‐dyskinetic potential of LCIG.

The strengths of this study include the use of real‐world data collected at routine clinical settings from a large, multinational cohort and an evaluation of outcome measures that are aligned with the current scientific literature for PD. However, the limitations of this study relate to its retrospective data collection and purely observational design, with many analyses having missing data. Furthermore, the analyses were not specifically limited to patients who had data available at both baseline and the study visit, though the sample sizes were similar at both time points for most assessments. As patients were not specifically randomized into the LCIG monotherapy, daytime monotherapy, and polytherapy groups, there is a potential risk of confounding factors that could bias the results. Notably, patients with more severe disease were more likely to receive polytherapy; nonetheless, all three groups experienced a significant reduction from baseline in dyskinesia severity. Because patients in this analysis were required to have had at least 12 months of LCIG treatment, these patients may not be fully representative of all patients who initiate LCIG, which limits the generalizability of the results. Another limitation was the assessment of dyskinesia severity and duration using UPDRS Part IV, rather than a more detailed and comprehensive scale such as the UDysRS. The UDysRS offers a more objective and thorough evaluation of dyskinesia and its associated disability by incorporating structured, clinician‐observed assessments of motor tasks and evaluation of dyskinesia across different body regions.^32^ While the UDysRS may have offered a more thorough and precise characterization of the dyskinesia burden experienced by patients, the decision to utilize the UPDRS Part IV was driven by practical considerations inherent in our study design. Baseline data were collected retrospectively, which precluded prospective objective evaluation of dyskinesia with the UDysRS. Secondly, at the time the study commenced, the UDysRS was not yet widely integrated into routine clinical practice. The analysis is also limited by the fact that very few patients with severely or completely disabling dyskinesias were included in the COSMOS study, which does not allow our analysis to draw generalizable conclusions for the effect (either beneficial or detrimental) of LCIG on the more severely affected group of dyskinetic patients.

This analysis provides real‐world data on the effectiveness of LCIG in improving dyskinesia in patients with advanced PD. LCIG led to a decrease in the duration and severity of dyskinesia, regardless of whether LCIG was administered as monotherapy or polytherapy. LCIG was also associated with improvements in dyskinesia severity, regardless of whether patients had high or low dyskinesia burden at baseline. Greater reductions in the duration of dyskinesia were observed in patients with high vs. low dyskinesia burden. These results confirm findings from previous studies^14^, ^22^ that LCIG is an important therapeutic option for managing dyskinesia among patients with advanced PD, particularly for those who would benefit from monotherapy treatment. In the era of personalized treatment and precision medicine, larger studies are needed to identify specific patient profiles that would most benefit from the anti‐dyskinetic effects of LCIG, as well as those that may not.

## Author Roles

(1) Research project: A. Conception, B. Organization, C. Execution; (2) Statistical Analysis: A. Design, B. Execution, C. Review and Critique; (3) Manuscript Preparation: A. Writing of the first draft, B. Review and Critique.

A.F.: 1C, 3B.

C.S.: 1C, 3B.

T.G.: 1C, 3B.

R.J.: 1C, 3B.

P.S.: 1C, 3B.

J.S.: 1C, 3B.

L.V‐D.: 1C, 3B.

M.S.: 1C, 3B.

L.B.: 1A, 1C, 3B.

A.S.: 1C, 2B, 2C, 3A, 3B.

J.C.P.: 1A, 1C, 2A, 2B, 2C, 3A, 3B.

N.K.: 1C, 3B.

## Disclosures

**Ethical Compliance Statement:** The study received approval from the independent ethics committees and/or institutional review boards at each investigator's institution and complied with applicable regional regulations. Before data collection, written informed consent was provided by each patient or legally authorized representative. We confirm that we have read the Journal's position on issues involved in ethical publication and affirm that this work is consistent with those guidelines.

**Funding Sources and Conflicts of Interest:** AbbVie funded this study and participated in the study design, research, analysis, data collection, interpretation of data, reviewing, and approval of the publication. In addition, AbbVie funded the medical writing support provided for this manuscript. All authors had access to relevant data and participated in the drafting, review, and approval of this publication. No honoraria or payments were made for authorship. AF has received compensation for serving as a consultant and speaker for AbbVie. CS has received honoraria for lecturing, advisory fees, educational grants, and travel grants from AbbVie and was an investigator on the DYSCOVER Study. TG was a study investigator and an advisor for AbbVie. She has received travel support for her team and herself from AbbVie. RJ was a study investigator and has received honoraria from AbbVie. PS was a study investigator and has received advisory fees from AbbVie. JS was a study investigator and has received compensation from AbbVie for consultancies and speaker activities. LV‐D was a study investigator and has received honoraria from AbbVie for delivering educational presentations and providing advice. AbbVie supported her travel to the International Congress of Parkinson's Disease and Movement Disorders in 2024. MS has received honoraria for lectures at symposia and consultant fees from AbbVie. LB, AS, and JCP are employees of AbbVie and may hold stock and/or share options. NK has received honorarium from AbbVie for lecturing at symposia. He has been a consultant for AbbVie and was an investigator on the DYSCOVER study.

**Financial Disclosures for the Previous 12 Months:** AF has received compensation as a consultant from Abbott, Boston Scientific, Medtronic, and UCB. He has received research support from Boston Scientific, Medtronic, Michael J. Fox Foundation for Parkinson's Research, and the University of Toronto. He has received honoraria for serving as a speaker from Boston Scientific, Chiesi, Medtronic, Novartis, Teva, and UCB. CS has received honoraria for lecturing, advisory fees, educational grants, and travel grants from ITF Hellas, Innovis, Merck Serono, and Teva. She received research funding from the Greek National Precision Medicine Network, the European Union's Horizon 2020 research, and the innovation program under the Marie Sklodowska‐Curie grant agreement No 101007926 and from the European Union's Horizon Europe program Excellence Hubs – HORIZON‐WIDERA‐2022‐ACCESS‐04‐01 under grant agreement No. 101087071. TG was a study investigator and an advisor for Medison, Neuroderm, Teva, and Truemed. She has received research support from the International Movement Disorders Society, Parkinson's Foundation, and travel support for her team and herself from Medison, Alphamedix, and Medtronic. RJ has received honoraria from Allergan, Cardion, Ipsen, and Medtronic for consultancies and lectures. PS has received advisory fees from Lundbeck and Takeda. He has received honoraria from Zambon for speaker activities. He has received research support from Knut and Alice Wallenberg Foundation, Stockholm City Council, Swedish Parkinson Fund, and the Swedish Brain Foundation. JS has received compensation from BI, Ever Pharma, GSK, KrKa, Lundbeck, Novartis, Pfizer, STADA, Teva, and UCB for consultancies and speaker activities. LV‐D has received honoraria from Bial, Esteve, and Zambon for delivering educational presentations and providing advice. MS has received honoraria for lectures at symposia and consultant fees from AOP Orphan, BI, KrKa, Merck, Novartis, Roche, Sanofi, Servier, Teva, and UCB. LB, AS, and JCP declare that there are no additional disclosures to report. NK has received honorarium from Abbott, Boston Scientific, GlaxoSmithKline, KrKa, MEdis, Medtronic, Richter Gedeon, and UCB for lecturing at symposia. He has been a consultant for Abbott, KrKa, and Teva, and has received research funding from Abbott; the Hungarian National Research, Development and Innovation Office; Medtronic; and University of Pécs.

## Data Availability

AbbVie is committed to responsible data sharing regarding the clinical trials we sponsor. This includes access to anonymized individual and trial‐level data (analysis data sets), as well as other information (eg, protocols, clinical study reports, or analysis plans), as long as the trials are not part of an ongoing or planned regulatory submission. This includes requests for clinical trial data for unlicensed products and indications. These clinical trial data can be requested by any qualified researchers who engage in rigorous, independent scientific research, and will be provided following review and approval of a research proposal and Statistical Analysis Plan (SAP) and execution of a Data Sharing Agreement (DSA). Data requests can be submitted at any time after approval in the United States and Europe and after acceptance of this manuscript for publication. The data will be accessible for 12 months, with possible extensions considered. For more information on the process or to submit a request, visit the following link: https://vivli.org/ourmember/abbvie/, then select “Home.”
